# Exploration of acupuncture therapy in the treatment of MCI patients with the ApoE ε4 gene based on the brain-gut axis theory

**DOI:** 10.1186/s12906-023-04060-2

**Published:** 2023-07-08

**Authors:** Yuanyuan Jin, Jin Chen, Qichen Chai, Jianfang Zhu, Xiaoqing Jin

**Affiliations:** 1 Department of Acupuncture and Moxibustion, Zhejiang Hospital, No. 1229 Gudun Road, Xihu District, Hangzhou, China; 2 Department of General Medicine, Zhejiang Hospital, No. 1229 Gudun Road, Xihu District, Hangzhou, China

**Keywords:** Mild cognitive impairment, Gut microbiota, *Apolipoprotein E* gene polymorphism, Acupuncture therapy, 16S rRNA sequencing

## Abstract

**Background:**

Mild cognitive impairment (MCI) is the predementia phase of Alzheimer’s disease (AD). The intestinal microbiome is altered in MCI and AD, and apolipoprotein E (*ApoE*) ε4 gene polymorphism is a risk factor for the progression of MCI to AD. This study aims to investigate the improvement in cognitive function of MCI patients with and without *ApoE* ε4 due to acupuncture and the changes in gut microbiota community composition and abundance in MCI.

**Methods:**

This randomized assessor-blind controlled study will enrol MCI patients with and without the *ApoE* ε4 gene (n = 60/60). Sixty subjects with the *ApoE* ε4 gene and 60 subjects without the *ApoE* ε4 gene will be randomly allocated into treatment and control groups in a 1:1 ratio. Intestinal microbiome profiles will be evaluated by 16 S rRNA sequencing of faecal samples and compared between the groups.

**Results/conclusions:**

Acupuncture is an effective method to improve cognitive function in MCI. This study will provide data on the relationship between the gut microbiota and the effectiveness of acupuncture in patients with MCI from a new angle. This study will also provide data on the relationship between the gut microbiota and an AD susceptibility gene by integrating microbiologic and molecular approaches.

**Trial registration:**

www.chictr.org.cn, ID: ChiCTR2100043017, recorded on 4 February 2021.

## Introduction

Mild cognitive impairment (MCI) is a clinical state between normal ageing and Alzheimer’s disease (AD) that does not meet the definition of dementia. It is characterized by a gradual decline in memory or other cognitive functions that does not affect activities of daily living (ADL) [1]. The prevalence of MCI increases with age, from 6.7% at 60–64 years to 25.2% at 80–84 years [2]. The prevalence of MCI in China in the population aged > 65 years old is 15.5% [1]. MCI constitutes a major health care burden for society, with the global cost in 2010 estimated at over 6 trillion US dollars [3].

Apolipoprotein E (ApoE) is a plasma protein that regulates cholesterol balance and the metabolism of lipoproteins in the nervous system [4]. The 3 isoforms of ApoE—ApoE2, ApoE3, and ApoE4—differ by 1 or 2 amino acid residues but have markedly different effects on AD risk: *ApoE2*, which has an allele frequency of ~ 8%, is known to be neuroprotective, whereas *ApoE4* has a prevalence of approximately 14% and is one of the strongest risk factors for AD [5–7]. The *ApoE* ε4 allele has been widely studied as a risk factor for MCI and AD [8, 9]. The lifetime risk of developing AD at the age of 85 years is 65% for individuals with 2 copies of the *ApoE* ε4 allele, compared with 10% for those with the ε3/ε3 genotype [10].

The aetiology of AD is multifactorial, and pathologic changes such as the accumulation of amyloid β and tau proteins occur years before the onset of signs and symptoms [11]. The *ApoE* ε4 allele has been implicated in AD pathogenesis [12], but multiple gene variants may have combinatorial effects on AD susceptibility. One study found that the presence of *ApoE* ε4 was associated with a lower Mini-Mental State Examination (MMSE) score but only in subjects with a substantial iron homeostasis gene burden [13].

In the management of dementia, early intervention in MCI is key [14]. Acupuncture is widely used to improve the clinical symptoms and delay the progression of MCI [15], as there are no appropriate drug treatments [2].

The complexity and multifactorial aetiology of AD pose unique challenges to studying its pathogenesis and developing treatment methods. Various pathological changes occur several years before the first appearance of symptoms [11]. Increasing evidence suggests that the gut microbiota is also involved in memory formation, processing, and storage [16]. Intestinal microorganisms are thought to contribute to the pathology of neurodegenerative diseases via the gut–brain axis [17–19] according to the observation that gut microbiota community composition and abundance are altered in patients with AD [18, 20, 21]. Intestinal flora can be readily isolated from faecal samples.

The study of intestinal flora and the gut–brain axis can potentially reveal novel diagnostic biomarkers for AD [17, 20, 22], but there is conflicting evidence regarding the relationship between the *ApoE* ε4 polymorphism and the gut microbiome profile. One study reported that differences in gut microbiota communities at the genus level between AD patients and normal subjects were not correlated with the *ApoE* genotype [20]. On the other hand, using the common voxel-based specific regional analysis system for AD scores, it was demonstrated that cortical and hippocampal atrophy was associated with changes in gut flora composition and abundance and *ApoE* polymorphism in MCI patients [23].

Although studies on the gut microbiota and acupuncture are lacking, the discovery of the partial relief of dysbiosis in the gut microbiome in MCI by acupuncture [24] has aroused great interest among researchers. Additionally, genetic markers that predict the progression of MCI are needed so that interventions to prevent AD development can be implemented. To this end, the proposed study will investigate the relationship between *ApoE* gene polymorphisms and the intestinal microbiome profile in patients with MCI. Exploring the evolution process of the intestinal microbiome of patients carrying different genes before and after acupuncture can provide more research data to reveal the relationship between the gut microbiota and neurodegenerative diseases.

## Methods

### Study design and population

**Fig. 1:**
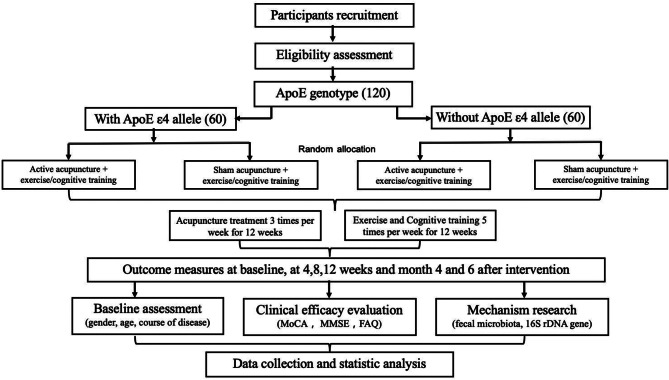
Study flow chart

This single-centre, randomized, assessor- and data analyst-blind controlled study will include 2 groups of patients—those with and without *ApoE ε4* gene polymorphism (n = 60/group)—recruited from the outpatient department of Zhejiang Hospital. Each group of patients will be randomly assigned to the acupuncture group or the sham acupuncture group. The study protocol was approved by the Ethics Committee of Zhejiang Hospital (2021-LS-12 K) on January 25, 2021. Each participant and/or their guardian will be informed of the nature of the study by telephone and will be required to sign the informed consent form before enrolment, which will take place from March 2021 to December 2022. Data will be analysed, and the results will be published in 2023. The proposal is depicted in Fig. 1.

### Inclusion and exclusion criteria

The patient inclusion criteria, as defined according to our previous research [25], are as follows: (1) confirmed diagnosis of MCI; (2) deficits in ≥ 1 cognitive domains as determined by objective examination; (3) Clinical Dementia Rating score of 0.5; (4) normal ADL score; (5) MMSE score ≥ 24 but ≤ 28 and Geriatric Depression Scale (GDS) score < 9; (6) stable and reliable caregivers; (7) aged ≥ 60 years; and (8) provision of informed consent. The exclusion criteria [25] are as follows: (1) age < 60 years; (2) diagnosed with any type of dementia; (3) comorbid acute and chronic cerebrovascular disease, encephalitis, Parkinson’s disease, leukoencephalopathy, and other central nervous system diseases; (4) hypothyroidism, vitamin B12 deficiency, depression, syphilis, and other conditions that may affect cognitive decline; (5) GDS score ≥ 9; (6) severe hearing and visual impairment or inability to complete neuropsychologic tests; (7) liver and kidney dysfunction or haematopoietic system disorder; (8) use of antibiotics and probiotics within 1 month before enrolment; and (9) inflammatory gastrointestinal diseases, irritable bowel syndrome, chronic constipation, chronic diarrhoea, *Helicobacter pylori* infection (untreated), and other diseases affecting the intestinal flora.

### Sample size estimation

Using GPower 3.1.9.6 to determine the sample size, a single-factor ANOVA was selected for prior analysis at the 4-group level, with a response size of 0.4, a test level of 0.05, a test efficacy of 0.9, a sample size of 96, and a 25% dropout rate. The final sample size was determined to be 120 people, with 30 people in each group, which was similar to that of other studies [20, 26, 27]. The necessary number of participants was recorded at baseline. Before the end of the enrolment period, more patients will be recruited to replace those who were excluded after baseline examinations.

### Intervention

On the basis of our previous research [25], participants assigned to the acupuncture group will receive acupuncture stimulation, exercise and cognitive training. Acupuncture will be performed by a senior acupuncturist. The fixed acupoints are EX-HN1 (Sishencong) and GV20 (Baihui). The adhesive pads will be pasted on the acupoint surface after skin disinfection. The acupuncture needles will be inserted through the adhesive pads approximately 1 cm into the skin and left in place for 30 min, performing small thrusts and rotations every 10 min.

Participants assigned to the control group will receive sham acupuncture stimulation, exercise and cognitive training. The sham acupoints (1 cm away from the acupoint) used for the control group are separate from conventional acupoints or meridians. The adhesive pads will be pasted on the sham acupoint surface, placebo needles with a blunt tip replacing traditional needles will be inserted through the adhesive pads without penetrating the skin, and no manipulation will be performed. The treatment course will be three times a week for 12 weeks.

The exercise and cognitive training measures, based on our previous research [25], will be analogous to those of the control group. Exercise training will include Tai Chi, square dance, ball games, aerobic gymnastics, jogging, and other aerobic exercises acceptable to patients (5 times a week for 12 weeks, 1 h/time). The first three cognitive training sessions will be performed by professional rehabilitation doctors and rehabilitation therapists, during which family members will be trained. The subsequent training will be completed by family members at home. At the 4th and 8th weeks, the rehabilitation doctors will decide whether to adjust the training program after assessment.

### Randomization

Participants will be randomly divided into treatment (active acupuncture + exercise/cognitive training) and control groups (sham acupuncture + exercise/cognitive function training) using the method of a random parallel controlled trial as in our previous research [25]. Qualified subjects will be numbered and grouped by the random grouping numbers generated by SPSS software to ensure balance between the two groups.

### Blinding

We will try to ensure that participants receive comparable treatment measures. All researchers and statisticians responsible for data recording will be blind to the randomization, as in our previous research [25].

### Clinical assessment

The patients will be examined and MCI will be diagnosed by a trained neurologist. Clinical information will be collected through patient interviews and a review of medical records and will include the following: age of MCI onset; history of MCI; onset type; number of episodes; and MMSE, Montreal Cognitive Assessment, and Functional Activity Questionnaire scores. We will also collect detailed information on defecation status, number of completely autonomous bowel movements per day, and stool characteristics (Bristol Stool Form Scale). Data on patients’ diets will be collected with the Food Frequency Questionnaire [28, 29]. The mental state and behaviour of patients with MCI will be evaluated. The GDS will be used to screen depression [30].

### Outcome measurements

Scales of clinical dementia rating (CDR, MMSE, MoCA) and ADL will be collected at baseline, during the treatment (weeks 4 and 8), at the end of the treatment, and at months 4 and 6 after the intervention.

#### Primary outcome

According to the research objectives, the improvement of cognitive function as assessed by MoCA will be regarded as the main result of the study. The MoCA covers a wide range of cognitive fields, including attention and concentration, executive function, memory, language, visuospatial structure skills, abstract thinking, calculation, and orientation. It is specifically designed to screen for MCI with high sensitivity (80–100%) and purposefulness (50–76%) [31]. The patient’s faeces will be stored in the refrigerator at − 80 °C. 16 S rDNA sequencing will be used to detect the changes in intestinal flora distribution in patients.

#### Secondary outcomes

Secondary measures will include the MMSE and Functional Activity Questionnaire (FAQ) scores. The MMSE has 11 questions and tests five aspects of cognitive function. The highest score is 30 points, and a score of < 24 points is regarded as abnormal dementia [32]. Daily abilities include basic activities of daily living (BADL) and instrumental activities of daily living (IADL). The former refers to the most basic abilities required for independent living, and the latter refers to complex daily or social activities. The diagnosis of MCI requires normal BADL and slight impairment of IADL. FAQ is a commonly used IADL evaluation scale that has been proven to distinguish between people with normal cognitive function and those with MCI (level II evidence) (Professional Committee of cognitive disorders, Chinese Medical Association, 2018 Chinese guidelines for diagnosis and treatment of dementia and cognitive impairment (V): diagnosis and treatment of mild cognitive impairment 2018) and can be used as an indicator of the transformation from MCI to AD (level II evidence) [33].

### Blood sampling and assay

Within a restricted time window (08:00–10:00), venous blood samples will be collected from participants in the fasting state. Genomic DNA will be extracted from leukocytes using a commercial kit (Wizard Genomic DNA Purification Kit; Promega, Madison, WI, USA; product no. A1120), and the V3/V4 hypervariable region of the *16 S* gene will be amplified by PCR using primers rs429358-rs7412-F1 (TCGGAACTGGAACAACT) and rs429358-rs7412-R1 (ACCTGCTCCTTCACCTCGT). The reaction will contain 1 µl each of forward and reverse primers (10 µmol/l), 25 µl of 2× EasyTaq PCR SuperMix, 2 µl DNA, and 21 µl ddH_2_O. The expected PCR product is 482 bp and will be resolved by agarose electrophoresis before confirmation by sequencing (Hangzhou Meredith Biotechnology Co., Hangzhou, China) on an ABI3730xl system (Applied Biosystems, Foster City, CA, USA) [34].

### Faecal microbiome analysis

The V3/V4 sequence will be analysed by a bioinformatics approach with an error correction protocol. Quality filtering, including pruning low-quality bases at the beginning and end of each read (https://github.com/najoshi/sickle), will be performed before assembling paired reads using Paired-End reAd mergeR v0.9.1 (http://www.exelixis-lab.org/web/software/pear), with Bayes Hammer used for error correction [35, 36]. The size selection filter and Basic Local Alignment Search Tool (BLAST: https://blast.ncbi.nlm.nih.gov/Blast.cgi) search will be set to the expected amplifier size.

Quantitative Insights Into Microbial Ecology 2 (http://qiime.sourceforge.net/) will be used to analyse corrected and assembled reads [30, 37]. De novo ultrafast sequence analysis and open reference operational taxonomic unit (OTU) clustering will be performed, and de novo chimaera detection will be conducted using UCHIME v5.1 (http://drive5.com/uchime) [38, 39]. Taxonomic classification of OTUs will be conducted using naive Bayesian RDP Release v11.5 (Ribosomal Database Project; http://rdp.cme.msu.edu/) and the Green genes classification taxonomic database (https://greengenes.secondgenome.com/).

The Bray–Curtis distance, which is based on count statistics of sortable OTUs [40], will be used to determine differences in β diversity, and principal coordinate analysis will be used for 3-dimensional visualization of the distance matrix. The significance of diversity differences between groups will be evaluated with the Adonis test, with a test level of α = 0.05.

Genus-level differences in faecal microbiota between MCI patients with and without the *ApoE* ε4 allele will be determined based on linear discriminant analysis (LDA) effect size (log linear LDA score > 2.0 and P value < 0.01).

### Data analysis

Measurement data will be expressed as the means ± standard deviations, and count data will be expressed as frequencies. Data will be analysed using SPSS Statistics for Windows v21 (IBM, Armonk, NY, USA). Pearson’s χ2 test or Fisher’s exact test will be used to analyse classified variables. The t test will be used if the normal distribution and homogeneity test are satisfied, and the rank sum test will be used for nonnormality or nonuniformity of variance. The Kruskal‒Wallis test will be used for multigroup comparisons, and the Wilcoxon rank sum test will be used for comparisons between two samples. For repeated measurement data, repeated measurement analysis of variance will be used. For the pairwise comparison of multiple groups of data, the Bonferroni method will be used to adjust the test level.

## Results

Enrolment began in March 2021, and the total number of participants at the time of this writing is 98 patients. The final results will be published in 2023.

## Discussion

MCI is the prophase state of dementia and the key phase for early intervention. At present, there is no drug that can be used in the MCI stage. Acupuncture reflects the value of early intervention in MCI. The purpose of this study is to evaluate the difference in the curative effect of acupuncture on MCI patients with and without the *ApoE ε4* gene polymorphism and to explore the relationship between the acupuncture mechanism and changes in the intestinal flora. We hope to be able to provide assessable biomarkers associated with acupuncture treatment.

*ApoE* ε4 is associated with degeneration of brain capillary pericytes that maintain blood–brain barrier (BBB) integrity [8, 41]. *ApoE* ε4 carriers (ε3/ε4 or ε4/ε4 alleles) are distinguished from noncarriers (ε3/ε3) by BBB breakdown in the hippocampus and medial temporal lobe [8]. Disruption of the gut flora balance in pathogen-free mice has been linked to increased BBB permeability in the embryonic stage of development and in adults [42] as well as central nervous system abnormalities such as cortical myelination, hippocampal neurogenesis, cognitive function, and memory formation [43]. It has been suggested that systemic inflammation induced by compounds secreted by bacteria impairs the BBB and promotes neurodegeneration [44–46]. We hypothesize that specific types of microbiota contribute to *ApoE* ε4-associated breakdown of the BBB in patients with MCI and can serve as biomarkers for progression to AD.

EX-HN1 (Sishencong) and GV20 (Baihui) have the local effect of regulating brain function. Acupoints can regulate the brain network by increasing connections between cognitive-related regions, thereby improving cognitive function in MCI patients [47]. By improving brain function, acupoints further affect the movement, sensation, and secretion of the gastrointestinal tract through the descending pathway of the gut–brain axis and regulate the composition and behaviour of the gut microbiota [48]. The purpose of our research is to elucidate the relationship between acupoints and gut microbiota.

Clinically, acupuncturists treat patients individually based on their symptoms. As the fixed acupuncture program can reduce research deviation and is more conducive to promotion, we selected fixed acupoints.

This study has some limitations. The study was designed as a single-centre pilot study due to the lack of adequate preliminary studies. Second, some possible interfering factors (pathogenic genes) have not yet been considered.

## Conclusion

This study will explore the relationship among gut microbiota, AD susceptibility genes and acupuncture. It also aims to show that acupuncture can regulate the intestinal flora while improving cognitive function.

## Data Availability

Not applicable.

## List of abbreviations

MCI: mild cognitive impairment
AD: Alzheimer’s disease
ApoE: apolipoprotein E
ADL: activities of daily living
MMSE: Mini-Mental State Examination
GDS: Geriatric Depression Scale
BLAST: Basic Local Alignment Search Tool
OTU: operational taxonomic unit
USEARCH: ultrafast sequence analysis
LDA: linear discriminant analysis
BBB: blood–brain barrier
